# Polyethylene Terephthalate Film-Based Sample Holder for Fixed-Target Serial Femtosecond Crystallography

**DOI:** 10.3390/ma19163449

**Published:** 2026-08-14

**Authors:** Jaehyun Park, Sehan Park, Ki Hyun Nam

**Affiliations:** 1 Pohang Accelerator Laboratory, Pohang University of Science and Technology, Pohang 37673, Republic of Korea; 2 College of General Education, Kookmin University, Seoul 02707, Republic of Korea

**Keywords:** serial femtosecond crystallography, fixed-target, sample holder, polyethylene terephthalate, PET

## Abstract

Serial femtosecond crystallography (SFX) using X-ray free-electron lasers (XFELs) enables the determination of macromolecular and chemical structures along with minimizing radiation damage. Sample delivery using the fixed-target (FT) scanning method minimizes both sample consumption and physical damage during delivery. When XFELs penetrate the FT sample holder, background scattering generated from the holder material can interfere with data processing, which consequently indicates the importance of selecting an appropriate FT sample holder material according to specific experimental conditions. We introduce a polyethylene terephthalate (PET) film-based sample holder that can be applied to data collection for both small-molecule and macromolecule XFEL diffraction studies. Background scattering analysis revealed that the PET film-based sample holder produced relatively low X-ray scattering in the high-resolution range (<10 Å) compared with a nylon mesh-based sample holder composed of polyimide and nylon mesh. Conversely, background scattering generated from the PET film was relatively higher in the low-resolution range (>10 Å) than that with the polyimide film; however, this low-resolution background was negligible for data processing. Thin PET films were manually perforated to promote the deposition of microcrystals in random orientations. The crystal samples were then deposited onto the films and sealed with an additional PET film to prevent dehydration. Using the PET film-based sample holder, we successfully collected diffraction data from barium titanate without unwanted background scattering from the sample holder. Furthermore, the room-temperature structure of glucose isomerase was determined using the PET film-based sample holder. Therefore, PET film-based sample holders can be applied to both small-molecule and macromolecular SFX data collection.

## 1. Introduction

Serial femtosecond crystallography (SFX), using X-ray free-electron lasers (XFELs), enables the determination of the crystal structure of biomolecules and chemical molecules along with minimizing radiation damage [1,2,3,4]. SFX with pump–probe experiments conducted using optical lasers allows time-resolved studies of the molecular dynamics of photoactive proteins or chemicals [5,6,7,8,9]. SFX with the mix-and-inject method allows the visualization of time-resolved intermediate states for enzyme reaction with substrate or the interaction between protein and inhibitor [10,11,12,13]. Furthermore, SFX diffraction data collection of protein crystals in the presence of the substrate allows tracing of the reaction state based on the partial electron density map [13]. Therefore, SFX provides a more accurate and detailed molecular structure and molecular mechanism for both macromolecules and chemical structures than traditional X-ray crystallography.

In the SFX experiment, a large number of microcrystals are delivered into the XFEL interaction point [14,15,16,17,18]. Each crystal is exposed to the XFEL only once, yielding partial diffraction information [19]. These partial diffractions are further merged to determine the complete three-dimensional structure [20,21,22,23]. Various crystal delivery methods have been developed for SFX experiments, including injectors [24,25], viscous-medium injectors [26,27,28], chemical mixing injector [11,29], fixed-target (FT) scanning [30,31,32], and hybrid approaches [33,34,35,36]. Among these, the FT scanning method has the advantage of minimizing mechanical stress on the crystals during sample delivery and dramatically reducing sample consumption compared with injector-based sample delivery methods [37,38].

To date, various FT sample holders, such as silicon chips [39], silicon nitride [30], Mylar films [40,41], viscous medium [42], nylon mesh [32], polyimide tubing [43], polyimide mesh [44], cyclic olefin copolymer [45], and parylene–N polymer [46], have been developed for serial crystallography (SX) experiments. In general, FT sample holders designed with regularly spaced holes allow crystals to be stably mounted in the holes along with minimizing background scattering during XFEL exposure [37,39]. Nevertheless, such devices require precise machining, extended fabrication time, and considerable effort. Meanwhile, sheet-on-sheet sandwich [40] or nylon mesh holders [32,47] can be fabricated rapidly and cost-effectively, using commercially available materials. In Mylar sheet-on-sheet holder, crystals were mounted between two 2.5 μm-thick Mylar films [40]. In contrast, the nylon mesh-based sample holder consists of a nylon mesh sandwiched between two 25 μm-thick polyimide films to prevent crystal sedimentation by gravity [32,47]. These film-based sample holders typically generate X-ray background scattering during XFEL exposure, originating from both the sample holder and crystal solution. Nevertheless, this background scattering is generally negligible for data processing and therefore widely used for determining macromolecular structures in SX data collection.

During the accumulation of powder diffraction peaks for the analysis of Debye–Scherrer rings in small-molecule SFX (smSFX) using a nylon mesh-based sample holder, we observed that the rings originated from both the target molecules and the sample holder (Supplementary Figure S1). These powder diffraction ring patterns originating from the sample holder interfere with data analysis and potentially reduce overall data quality. Therefore, it is essential to develop sample delivery systems that minimize unwanted diffraction features, such as the Debye–Scherrer rings originating from FT sample holder materials, for improving data processing efficiency and achieving high-quality diffraction data.

Here, we introduce an application of a polyethylene terephthalate (PET) film-based sample holder designed to minimize unwanted scattering rings from the sample holder material for small-molecule SFX (smSFX) data collection. The thin PET film significantly reduces X-ray background scattering from the sample holder material at high resolution. Using this sample holder, we successfully collected both small-molecule and macromolecular diffraction data. This sample holder will be useful for SFX data collection for both chemical compounds and macromolecules.

## 2. Materials and Methods

### 2.1. Materials

The following materials were used in this study: PET film (catalog no. LP00013728, thickness: 2.5 μm; Goodfellow, Huntingdon, UK), nylon mesh (catalog no. NY4100010, thickness: 50 μm; MilliporeSigma, Burlington, MA, USA), polyimide film (Kapton^®^ HN, type 100HN, thickness: 25 μm; DuPont, Wilmington, DE, USA), BTO (BaTiO_3_: catalog no. 208108; Sigma-Aldrich, St. Louis, MO, USA), and GI (glucose isomerase: catalog no. HR7-102; Hampton Research, Aliso Viejo, CA, USA).

### 2.2. Preparation of the PET Film-Based Sample Holder

The PET film for crystal settling was manually punctured with the sharp tip of a syringe needle to increase film roughness and promote random orientation of the settled crystals. Two PET film frames were prepared by attaching the PET film to an aluminum window frame (20 × 20 mm, 300-μm thickness) using a double-sided adhesive tape. The punctured PET film was placed on the PET film frame, and the BTO or GI crystal suspensions (30 μL) were loaded onto the punctured PET film and spread evenly using a pipette tip. The crystal samples were then covered with a second PET film frame using a double-sided adhesive tape.

### 2.3. X-Ray Transmission Calculation

The X-ray transmission of the PET films was calculated using the CXRO database (http://henke.lbl.gov/optical_constants/; accessed on 8 March 2026).

### 2.4. XFEL Data Collection

SFX data collection was performed at the Nano Crystallography and Coherent Imaging (NCI) experimental end station [48] at Pohang Accelerator Laboratory X-ray Free-Electron Laser (PAL-XFEL) [49]. The XFEL wavelength and repetition rate were 1.3045 Å (9503 eV) and 30 Hz, respectively, with a photon flux of approximately 2–5 × 10^11^ photons/pulse. The XFEL beam size was focused to approximately 3 × 3 μm (vertical × horizontal, FWHM) using a Kirkpatrick–Baez (KB) mirror [50]. All diffraction data were collected at 23 °C ± 0.5 °C and recorded on an MX225-HS detector (Rayonix, LLC, Evanston, IL, USA) with 30 Hz readout.

### 2.5. Background Scattering Analysis

Single-shot XFEL data were collected using polyimide film (25 μm, 2 sheets), polyimide film combined with a nylon mesh (50 μm thickness), and PET film (2.5 μm, 2 sheets). The background scattering intensities of the sample holder materials were examined and visualized using ADXV (https://www.scripps.edu/tainer/arvai/adxv.html; accessed on 26 February 2026). The average intensity profiles were measured from the beam center to 1.8. Å using 10 images.

### 2.6. Diffraction Data Collection

The PET film-based sample holder containing BTO or GI crystals was mounted on a translation stage located on the XFEL focal point. The sample holder was raster-scanned at 50 μm intervals horizontally and vertically, with a translation speed of 1.5 mm/s applied in both directions. The hit rate and diffraction patterns were monitored using OnDA [51].

### 2.7. XFEL Data Processing

Data processing for GI was conducted at the Global Science Experimental Data Hub Center at the Korea Institute of Science and Technology Information [52]. Hit images containing Bragg peaks were filtered using Cheetah with the peakfinder8 algorithm [53]. The diffraction images were processed using CrystFEL (version 0.9.1+886ae521) [20] with the XGANDALF [54] indexing algorithm. The crystal-to-detector distance was determined based on the Gaussian distribution of the unit cell parameters [55]. Detector geometry was optimized using geoptimiser [56]. The indexed diffraction patterns were scaled, post-refined, and merged using partialator in CrystFEL.

### 2.8. Structure Determination

Molecular replacement (MR) was performed using Phaser-MR implemented in PHENIX (version 1.17.1) [57], with the crystal structure of GI (PDB code: 8WDH) [58] used as the search model. Structure building was accomplished using COOT (version 0.9.8.96) [59]. Structural refinement was conducted using phenix.refine in PHENIX, and water molecules were added during refinement with default parameter. The final model structure was validated using MolProbity [60]. Structural figures were generated using PyMOL (version 2.5.10) (http://pymol.org; accessed on 13 April 2026).

## 3. Results

### 3.1. Material Selection and X-Ray Background Scattering Analysis

We conducted smSFX data collection for BTO using a nylon mesh sample holder. During *d*-spacing analysis of the accumulated Debye–Scherrer rings of BTO, we detected unexpected scattering rings at approximately 3.75, 4.30 and 14.0 Å, which have not been previously reported in the *d*-spacing of BTO powder diffraction patterns (Supplementary Figure S1). Analysis of these scattering rings revealed that they were identical to previously reported X-ray background scattering, arising from nylon mesh and polyimide materials used in sample holders [32]. The weak background scattering generated from polyimide and nylon mesh in FT sample holders is negligible for SFX data processing [61,62,63]. Nevertheless, when diffraction patterns are accumulated in smSFX experiments, the background scattering generated from polyimide and nylon mesh can cause misinterpretation during *d*-spacing analysis or require additional computational work to mask unwanted diffraction rings generated from the sample holder materials.

**Figure 1:**
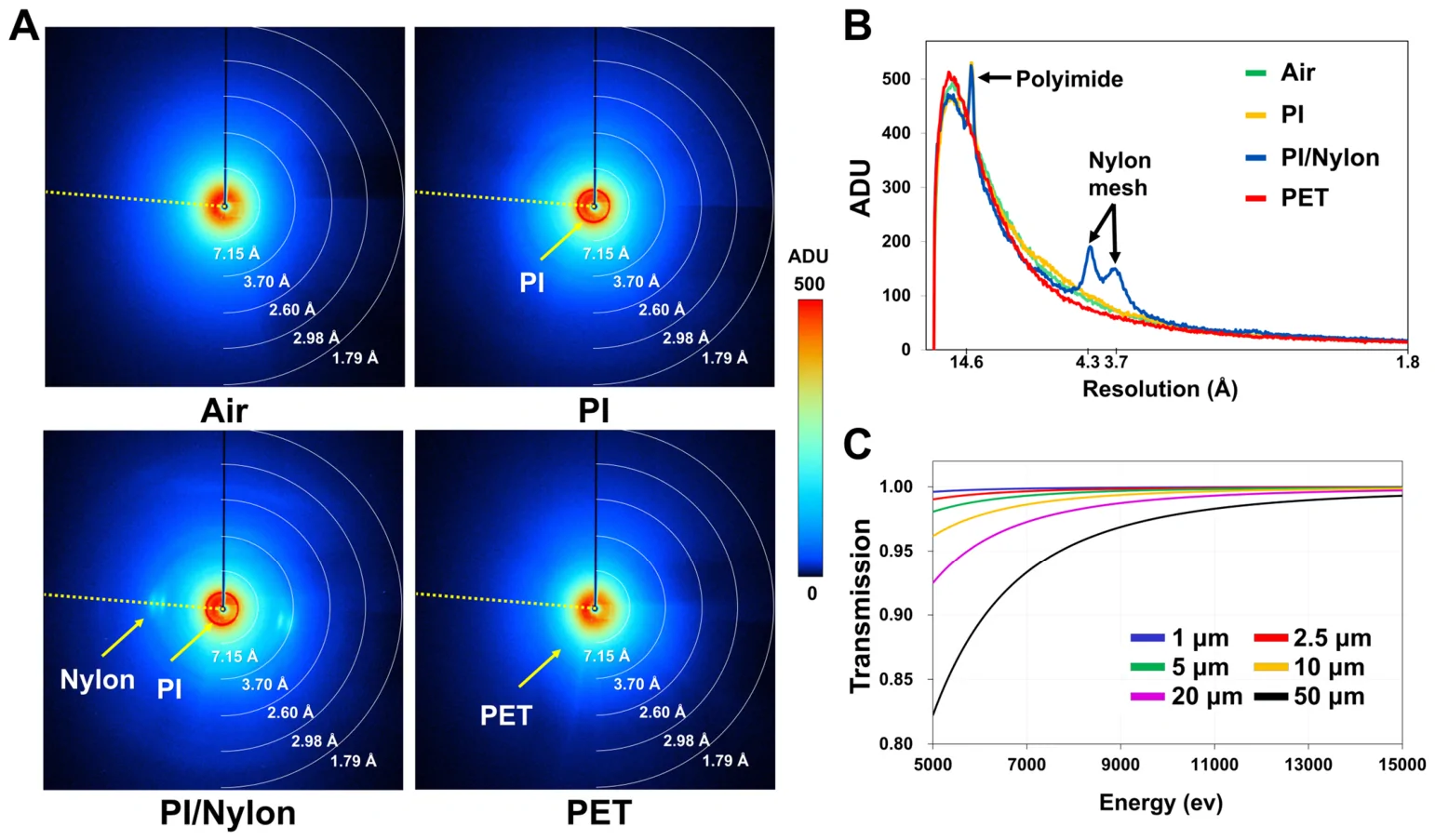
Analysis of XFEL background scattering from FT sample holder materials. (**A**) XFEL scattering images of air, polyimide (PI) film, PI film with nylon mesh (PI/nylon), and PET film. XFEL scattering generated from polyimide, nylon mesh, and PET films is indicated by yellow arrows. (**B**) 2D profile of background scattering intensities when X-rays are exposed to air (green), PI film (yellow), PI film with nylon mesh (blue), and PET film (red). X-ray scattering intensity was measured from the beam stopper to the resolution corresponding to 1.8 Å. The measurement region is indicated by the yellow dotted line in Figure 1A. The total thicknesses of the PI and PET films were 50 and 5 µm, respectively. (**C**) Calculation of X-ray transmission through a PET film (chemical formula: C_10_H_8_O_4_; density: 1.4 g/cm^3^) depending on film thickness over the energy range of 5–15 keV.

To avoid misinterpretation in data analysis and reduce background scattering, we considered the application of thin PET films that exhibit high mechanical strength and low water absorption, thus making them suitable for applications requiring structural stability and environmental isolation [64,65]. To determine whether the PET film is a suitable material for sample holders, we examined the XFEL scattering intensities and compared them with those obtained by exposing XFEL to air, polyimide film, and polyimide film with nylon mesh (Figure 1A and Supplementary Figure S2). In this study, a 2.5 μm-thick PET film was selected because it was commercially available and provided sufficient mechanical stability for sample handling and preparation. When XFEL was exposed to air without a sample holder, air scattering of ~480 ADU was observed in the 20–50 Å range, with the intensity decreasing toward higher resolution (Figure 1A). The polyimide film exhibited a diffraction peak of >500 ADU at ~14 Å, whereas the overall intensity trend was similar to that of air scattering (Figure 1B). The polyimide film with nylon mesh demonstrated background scattering peaks of >120 ADU at approximately 4.30 and 3.75 Å resolution, generated from the nylon material, in addition to the polyimide diffraction peak at ~14 Å (Figure 1B). The PET film exhibited no significant background scattering, such as those observed from the polyimide film or nylon mesh, in the high-resolution region (>10 Å); however, it demonstrated background scattering of ~10 ADU higher than that of air scattering in the low-resolution region (20–50 Å) (Figure 1B). The average scattering intensities of polyimide, polyimide/nylon, and PET film were approximately 108.8, 112.9, and 101.4 ADU, respectively (Supplementary Figure S3), indicating that the PET film provided relatively lower background scattering than the polyimide film or polyimide film with nylon mesh across most resolution ranges, except in the low-resolution region.

The X-ray transmission of PET films, which was calculated as a function of thickness (1–50 μm) (Figure 1C), tended to be lower at lower X-ray energies than at higher energies and exhibited a decreasing trend with increasing film thickness. For PET films with thicknesses of 1 and 2.5 μm, the X-ray transmission was >99% over the energy range of 5–15 keV (Figure 1C). For films thinner than 10 μm, the transmission remained at >96% across the entire energy range (Figure 1C). At 9.7 keV, which is commonly used for SFX data collection at PAL-XFEL; PET films thinner than 10 μm showed transmission values of >99%, whereas 20- and 50-μm-thick films exhibited transmission values of more than 98.9% and 97.5%, respectively (Figure 1C). Despite the relatively small impact of PET film thickness on X-ray transmission in the high-energy region, thinner PET films are preferable when considering background scattering intensity.

### 3.2. Fabrication of the PET Film-Based Sample Holder

Crystal deposition between Mylar films without a chip using the previously reported sheet-on-sheet sandwich method was considered feasible for SFX experiments [40]. Nonetheless, when crystal samples with specific shapes are deposited onto a flat film, they may preferentially settle on the surface in orientations determined by their morphology [42], which makes it difficult to collect a complete diffraction dataset or requires the collection of a larger number of images than conventional data collection [42]. Hence, promoting random crystal orientations on the film is critical for efficient data collection and processing. Previously, irregular crystal-mounting holes were produced on polyimide films using a laser-drilling method, which successfully increased the randomness of crystal orientation and enabled high-quality diffraction data collection [44]. Nevertheless, this approach requires specialized equipment, considerable effort, and high costs.

**Figure 2:**
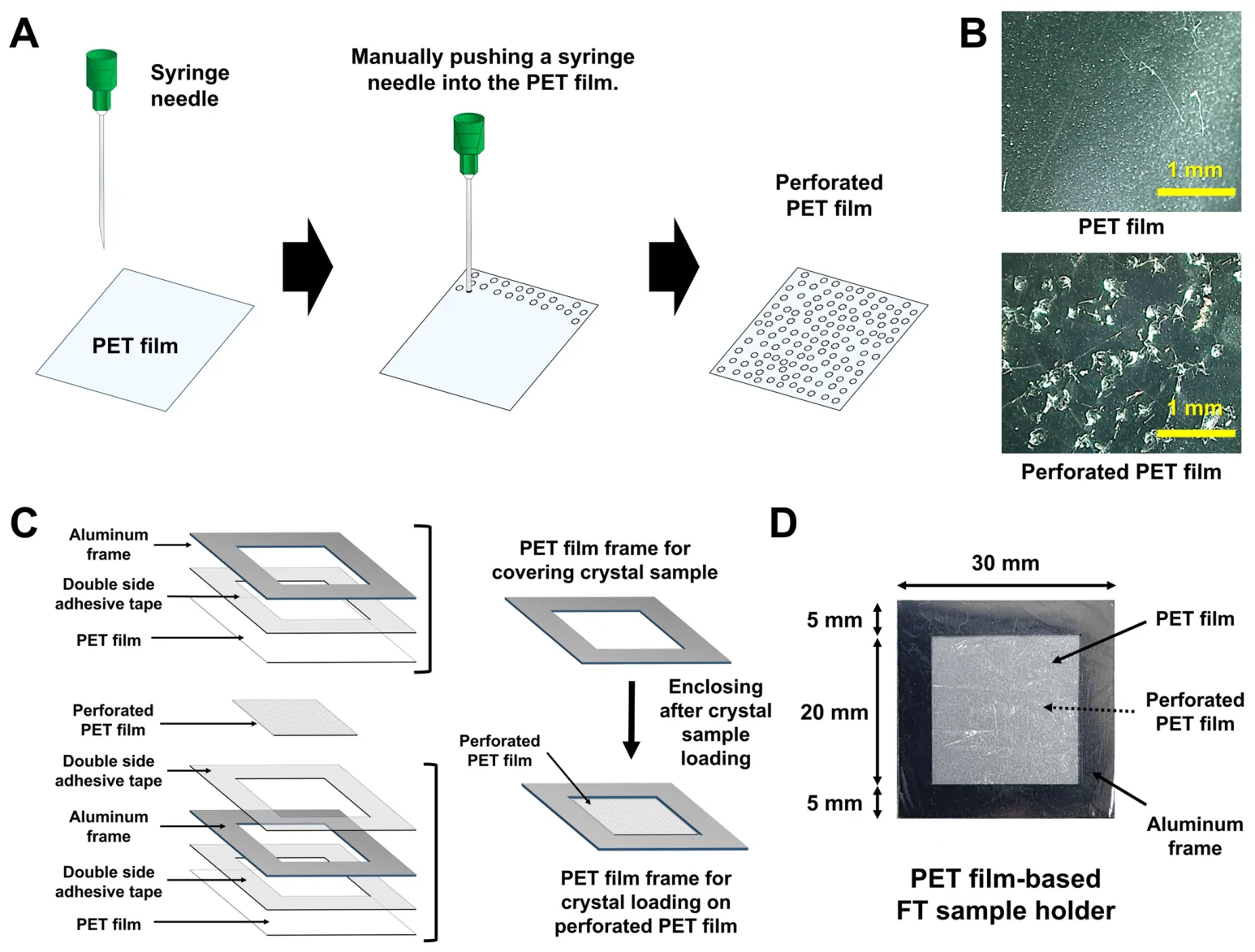
Preparation of the PET film-based sample holder. (**A**) Preparation of the perforated PET film using a syringe needle tip to promote random crystal orientation. (**B**) Microscope images of (top) an intact PET film used for crystal sample enclosure and (bottom) a perforated PET film produced by puncturing with a syringe needle tip to promote random orientation during crystal settling. (**C**) Schematic of the assembly of the PET film-based sample holder. (**D**) Photograph of the PET film-based sample holder for FT SFX data collection at PAL-XFEL.

Therefore, in the present study, to increase the random orientation of crystals on the PET film with minimal cost and effort, we manually perforated PET films across the entire surface using the sharp tip of a syringe needle (Figure 2A). Because of the manual perforation, both the size and shape of the holes were irregular (Figure 2B), which promotes the settling of more randomly oriented crystals than that with plain PET films. When crystal suspensions deposited on the perforated PET film were exposed to air during data collection, evaporation of the crystallization solution occurred. This can result in crystal dehydration, which can affect the unit cell parameters and space group and reduce diffraction quality [66]. Therefore, it is essential to maintain a hydrated environment during data collection. To prevent evaporation of the crystal solution, the crystals on the perforated PET films were enclosed between two PET films attached to an aluminum frame (Figure 2C), as previously reported for the fabrication of the nylon mesh-based sample holder [32].

During sample preparation, the perforated PET film was placed on a PET film attached to an aluminum frame (Figure 2C,D). The crystal suspension was transferred onto the perforated PET film, and the crystal samples were spread across the perforated surface using a pipette tip and then enclosed using a second PET film attached to an aluminum frame to prevent evaporation of the crystal solution (Figure 2C). Because the frame of the PET film-based sample holder consists of aluminum (Figure 2D), this sample holder can be easily mounted on or detached from the magnet at the FT sample holder position connected to the FT translation stage. The size of the aluminum frame of the PET film-based sample holder was 30 mm × 30 mm, with an inner empty area of 20 mm × 20 mm for sample loading on the perforated PET film (Figure 2D). When raster scanning was performed at 20, 30, and 50 μm intervals in both vertical and horizontal directions, 1,000,000, 444,443, and 160,000 diffraction images could be collected, respectively [38].

### 3.3. Application of the PET Film-Based Sample Holder for Barium Titanate

**Figure 3:**
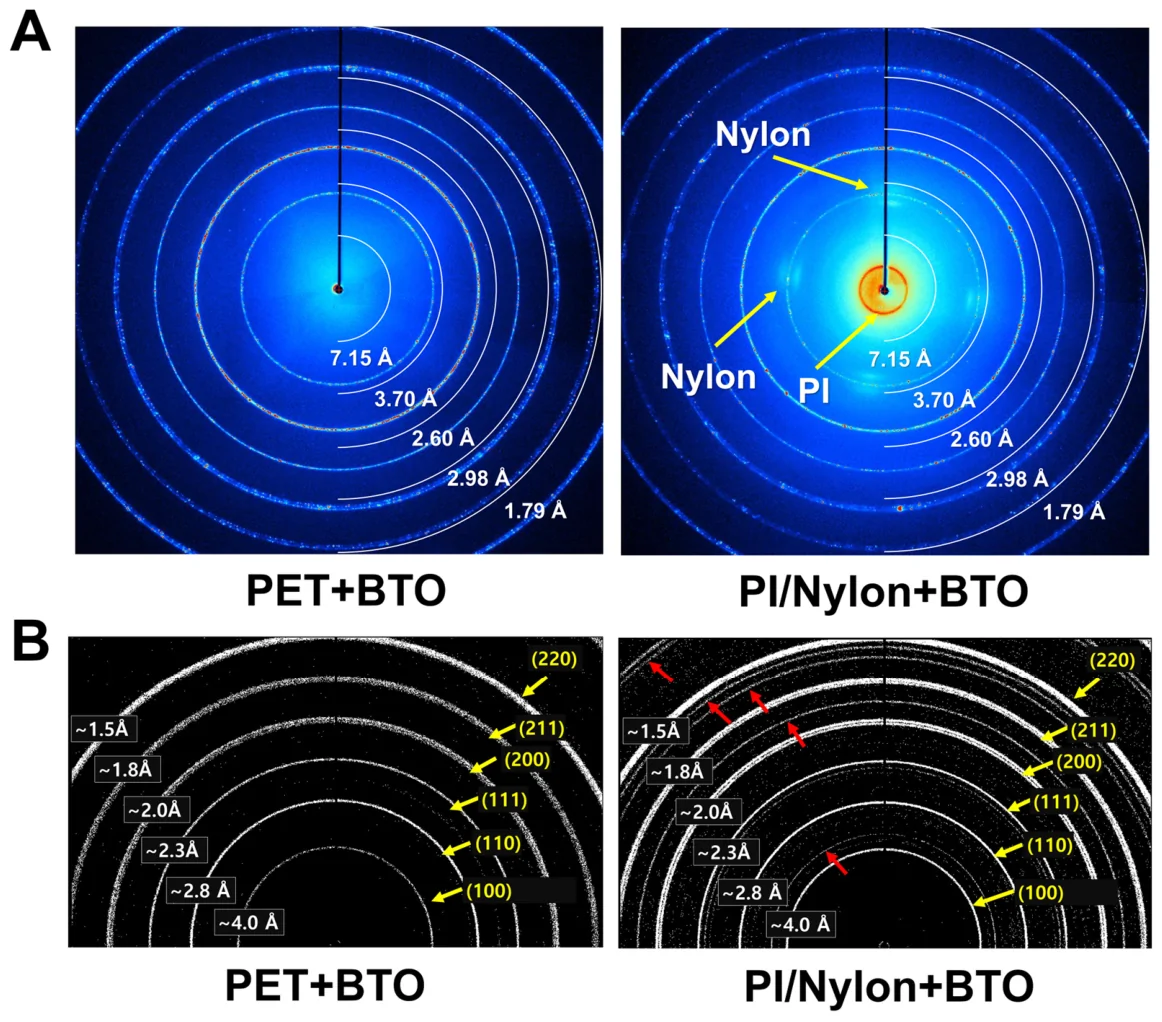
Application of PET film-based sample holder for BTO data collection. (**A**) Single-shot images of BTO powder diffraction patterns obtained using polyimide with PET film- and a nylon mesh-based sample holders. (**B**) Accumulated diffraction peaks of BTO delivered using a nylon mesh-based sample holder and a PET film-based sample holder. The BTO diffraction rings and unwanted diffraction ring patterns are indicated by yellow and red arrows, respectively.

To determine whether the PET film-based sample holder can be applied to the analysis of accumulated small-molecule diffraction peaks such as Debye–Scherrer rings, the XFEL diffraction data of BTO were collected using the PET film-based sample holder and compared with data collected using the nylon mesh-based sample holder. The single-shot diffraction patterns of BTO, obtained using the PET film-based sample holder, demonstrated clear powder diffraction rings with no significant background scattering originating from the PET film (Figure 3A). In contrast, the single-shot diffraction patterns, collected using the nylon mesh-based sample holder, also displayed clear powder diffraction rings of BTO but with relatively high background scattering originating from the polyimide film (~14 Å) and partial scattering originating from the nylon mesh (~3.8 and 4.3 Å) (Figure 3A). Moreover, the accumulated diffraction peaks of BTO, obtained using the PET film-based sample holder, showed well-defined powder diffraction rings without significant background scattering. The Debye–Scherrer rings demonstrated clear reflections of BTO corresponding to the (100), (110), (111), (200), (211), and (220) planes at approximately 4.0, 2.8–2.9, 2.3–2.4, 2.0, 1.6–1.8, and 1.4–1.5 Å, respectively (Figure 3A). Conversely, the accumulated diffraction peaks obtained using the nylon mesh-based sample holder exhibited several unwanted diffraction rings originating from the nylon mesh or polyimide, although the BTO diffraction rings were clearly observed (Figure 3A). Furthermore, additional Debye–Scherrer rings not attributable to nylon or polyimide were also detected, which are probably attributed to impurities in the sample holder. These findings indicate that the PET film-based sample holder is advantageous for the high-resolution *d*-spacing analysis of accumulated small-molecule diffraction data.

### 3.4. Application of PET Film-Based Sample Folder for Glucose Isomerase

The PET film-based sample holder was originally designed to minimize background scattering from the sample holder in smSFX experiments at high resolution; however, it can also be applied to macromolecular SFX data collection. Hence, to demonstrate the application of the PET film-based sample holder, we collected XFEL data for GI using this holder. During XFEL data collection using the PET film-based sample holder, GI crystals were stored in the PET sandwich for more than 2 h before data collection. The experimental setup and XFEL data collection were then performed for more than 40 min using a single PET film-based sample holder. GI crystal diffraction patterns were successfully collected throughout the entire data collection period, indicating that the crystals remained stable within the PET sandwich. In addition, no salt crystal diffraction peaks, which can be generated by dehydration of the crystallization solution, were observed in the XFEL images, suggesting that no significant dehydration of the crystallization solution occurred during XFEL data collection. Within 25 min, 40,879 images were obtained, and 15,113 hit images were filtered with a hit rate of 36.97%. The diffraction images of GI revealed no significant background scattering generated from the sample holder (Supplementary Figure S4). A total of 15,514 diffraction patterns were indexed, a number higher than the hit images, indicating that some diffraction images contained multiple crystal hits. The data were processed to a resolution of 1.65 Å, with completeness, signal-to-noise ratio (SNR), CC1/2, CC*, and R_split_ values of 100% (outer shell [1.71–1.65 Å]: 100%), 4.07 (0.83), 0.9069 (0.5108), 0.9752 (0.8223), and 24.52 (105.01), respectively (Table 1).

**Figure 4:**
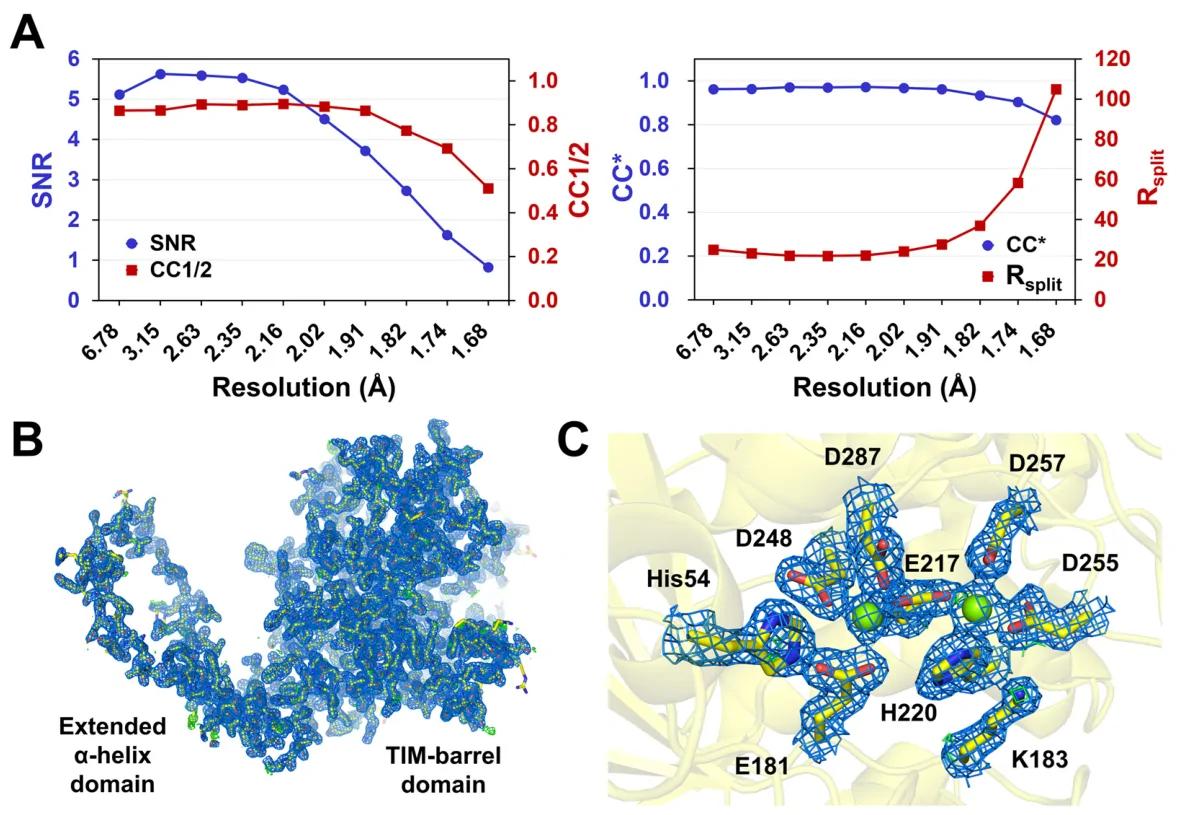
Application of the PET film-based sample holder for GI data collection. (**A**) Data processing statistics for SNR, CC1/2, CC*, and R_split_ across resolutions. 2mFo-DFc (blue, 1.0 σ) and mFo-DFc (green, 3.0 σ and red, −3.0 σ) electron density maps of (**B**) GI and (**C**) close-up view of the active site of GI.

Although no significant X-ray background scattering from the PET film was observed, the PET film can theoretically generate X-ray scattering from the material itself. To determine whether such background scattering affects data quality, we analyzed the SNR, CC, and R_split_ values across various resolution ranges (Figure 4A). We observed no significant impact on the high-resolution region, with no decrease in the statistical values. The XFEL structure of GI was determined with R_work_ and R_free_ values of 0.1956 and 0.2291, respectively. The electron density map clearly revealed almost all amino acid residues for both the TIM-barrel domain and extended α-helix domain, as well as the two metal-binding sites at the active site of GI (Figure 4B,C). The structural comparison of GI obtained using the PET film-based sample holder demonstrated high similarity with the previously reported GI structures delivered using the nylon mesh-based sample holder (PDB code: 6IRK, and 7DFJ) and gelatin-based crystal support (6LL2) determined by SX, with an r.m.s. deviation of 0.095–0.274 Å (Supplementary Figure S5). These data indicate that the PET film-based sample holder can be applied to XFEL data collection for macromolecules.

## 4. Discussion

The selection of appropriate sample delivery methods tailored to the crystal samples and research objectives is crucial in SFX experiments. Furthermore, when XFEL radiation passes through an FT sample holder, the properties of the materials comprising the holder are also important because background scattering generated by the holder can impact data quality.

In previously reported FT-SFX studies, a large number of diffraction images were collected, and each image was analyzed independently. Under these conditions, background scattering from the sample holder was generally negligible for data processing. At PAL-XFEL, nylon mesh-based sample holders have been widely applied in FT-SFX experiments for both small-molecule and macromolecular structure determination. In the present study, we found that when diffraction peaks are accumulated for analyzing Debye–Scherrer rings from chemical compounds, the accumulated background scattering generated from nylon mesh-based sample holders interferes with data processing. These results indicate that the suitability of conventional sample holder materials should be re-evaluated for accumulated diffraction experiments.

Although the Debye–Scherrer rings generated from the sample holder can be excluded during data processing by subtracting or masking these unwanted diffraction rings, additional data processing for subtracting the background scattering is not efficient or may result in the loss of useful diffraction information. Moreover, the overlap between Debye–Scherrer rings from the sample holder and diffraction peaks from the target molecule can lead to inaccurate data processing and a reduction in data quality, including the SNR. Therefore, it is essential to minimize background scattering from the sample holder for an accurate data analysis of the accumulated Debye–Scherrer rings or diffraction peaks from target molecules.

Among FT sample holders designed using film-enclosing approaches, background scattering can be reduced by either decreasing the thickness of the supporting material or using alternative materials with lower intrinsic scattering. In our initial diffraction experiment, the polyimide film and nylon mesh were approximately 25 and 50 μm thick, respectively. Although reducing the thickness of these materials can effectively decrease background scattering, thin polyimide films are commercially available but expensive, and nylon meshes with reduced thickness are not readily available. 

As an alternative to conventional materials, we used a thin PET film that is cost-effective and readily available. The XFEL background scattering analysis demonstrated that thin PET film-based sample holders produced minimal background scattering compared with nylon mesh-based sample holders, particularly in the high-resolution region, indicating their suitability for smSFX data collection by enabling the accumulation of diffraction peaks. Meanwhlie, the crystallinity and processing conditions of the PET film used in this study could not be confirmed. Because the diffraction characteristics of PET may vary depending on its crystallinity and processing conditions, the results presented here are limited to the specific PET film used in this study. Future studies using PET films with different crystallinities and processing conditions will be required to systematically evaluate their effects on background scattering.

The PET film-based sample holder is fundamentally similar to the previously reported Mylar sheet-on-sheet holder in that crystals are mounted between two PET films. However, in the present study, a manually perforated PET film was additionally used to promote random crystal orientation. This manual perforation of the PET film produces variations in hole size, density, and spacing, and these parameters cannot be reproduced exactly across sample holders. However, the resulting randomly perforated film surface is advantageous for promoting random crystal settling and orientation. Therefore, precise reproducibility of the perforation pattern is not considered a critical requirement for SFX data collection or processing. Nevertheless, perforating large areas of a PET film using a single syringe needle is labor-intensive. Therefore, to reduce the fabrication time and effort, the use of multiple needles or the development of a specialized tool capable of producing numerous perforations simultaneously could be considered for future improvements. Meanwhile, although the perforated PET surface was theoretically designed to promote random crystal orientation, the orientation distribution was not quantitatively evaluated in this study. Future studies comparing the diffraction data completeness obtained using perforated and non-perforated PET films will provide a more quantitative evaluation of the effect of the perforated surface on crystal orientation.

In this study, we compared the PET film-based sample holder with nylon mesh- and polyimide film-based sample holders that are routinely used at PAL-XFEL. However, a variety of materials, including Mylar, cyclic olefin copolymer, and other polymer-based supports, have also been applied to FT-SFX. Future systematic comparisons between PET film and these commonly used support materials under identical experimental conditions will provide a more comprehensive evaluation of their relative advantages and limitations for FT-SFX.

Meanwhile, in this study, the 2.5 μm PET film enabled successful sample delivery for SFX; however, its thinness occasionally made it susceptible to wrinkling during sample holder preparation. Therefore, careful handling and proper fixation of the PET film covering the crystals were required to maintain a flat sample holder surface. Furthermore, future studies should evaluate the radiation resistance of PET films and their compatibility with a wider range of crystallization solutions to facilitate their broader application in FT-SFX.

The X-ray transmission analysis of PET films demonstrated that they exhibited high XFEL transmission (>97.5%) across a thickness range of 1–50 μm in the high-energy region (>9.7 keV). However, as the film thickness increases, the background scattering from the PET film also increases. Therefore, using thinner PET films may exert less impact on data quality, including the SNR and data processing. Meanwhile, we theoretically evaluated the X-ray transmission of PET films with different thicknesses. To provide useful information for determining the optimal PET film thickness for accumulated diffraction data collection, further experimental evaluation of X-ray background scattering using PET films with various thicknesses is needed.

Approaches using sample holders with regular arrays of holes for crystal mounting allow systematic scanning of each hole with the XFEL beam and can minimize background scattering from the holder. Nevertheless, these methods require precise synchronization of the XFEL beam with the hole positions in the sample holder, which demands additional effort and increases the required beam time. Conversely, the PET film-based sample holder is composed of an X-ray-transparent material and does not require alignment with specific positions during data collection. Therefore, FT-SFX data collection using a PET film-based sample holder improves data collection efficiency without XFEL alignment.

The primary objective of this study was to reduce the background scattering observed from the nylon mesh-based sample holder during accumulated diffraction data collection for small-molecule crystals by applying a thin PET film-based sample holder. However, because different thicknesses of PET and polyimide films were used in this study, the background scattering originating from the two film materials cannot be directly compared. Future studies using PET and polyimide films with identical thicknesses are required to determine whether the reduced background scattering observed in this study originates primarily from the film material or from the reduced film thickness.

## 5. Conclusions

In this study, we introduced a PET film-based sample holder for FT-SFX data collection and successfully demonstrated its applicability to both small-molecule and macromolecular data collection. This sample holder is applicable for the accumulation of diffraction peaks from target molecules and for general FT SFX experiments for both small-molecule and macromolecular structure determination.

## Figures and Tables

**Table 1:** Data collection and structure refinement statistics of GI.

Data Collection	GI
Energy (eV)	9503
Space group	I222
Cell dimensions	
a, b, c (Å)	93.73, 99.53, 102.49
α, β, γ (°)	90.0, 90.0, 90.0
No. collected diffraction images	40,879
No. of hits	15,113
Diffraction patterns	15,514
No. of unique reflections	58,242 (5759)
Resolution (Å)	71.94–1.65 (1.71–1.65)
Completeness	100.0 (100.0)
Redundancy	436.9 (147.4)
I/σ(I)	4.07 (0.83)
R_split_ (%)	24.52 (105.01)
CC1/2	0.9069 (0.5108)
CC*	0.9752 (0.8223)
Wilson B factor (Å^2^)	29.10
Refinement statistics	
Resolution (Å)	71.40–1.65
R_work_/R_free_ (%)	0.1956/0.2291
B-factor (Å^2^)	
Protein	23.58
Metal	19.76
Water	34.58
R.m.s. deviations	
Bond lengths (Å)	0.005
Bond angles (°)	0.822
Ramachandran plot (%)	
favored	96.60
allowed	3.14
outlier	0.26
PDB code	9XEY

## Data Availability

The structure factor and coordinates of GI were deposited at the Protein Data Bank (https://www.rcsb.org) under access code 9XEY (https://doi.org/10.2210/pdb9XEY/pdb).

## Supplementary Materials

The following supporting information can be downloaded at https://www.mdpi.com/article/10.3390/ma19163449/s1.

## Abbreviations

The following abbreviations are used in this manuscript:

SFX	Serial femtosecond crystallography
XFEL	X-ray free-electron laser
PAL	Pohang Accelerator Laboratory
BTO	Barium titanate
GI	Glucose isomerase

## References

[1] Barends T.R.M., Stauch B., Cherezov V., Schlichting I. (2022). Serial Femtosecond Crystallography. Nat. Rev. Methods Primers.

[2] Chapman H.N., Caleman C., Timneanu N. (2014). Diffraction before Destruction. Philos. Trans. R. Soc. B.

[3] Weinert T., Olieric N., Cheng R., Brünle S., James D., Ozerov D., Gashi D., Vera L., Marsh M., Jaeger K. (2017). Serial Millisecond Crystallography for Routine Room-Temperature Structure Determination at Synchrotrons. Nat. Commun..

[4] Boutet S., Lomb L., Williams G.J., Barends T.R.M., Aquila A., Doak R.B., Weierstall U., DePonte D.P., Steinbrener J., Shoeman R.L. (2012). High-Resolution Protein Structure Determination by Serial Femtosecond Crystallography. Science.

[5] Tenboer J., Basu S., Zatsepin N., Pande K., Milathianaki D., Frank M., Hunter M., Boutet S., Williams G.J., Koglin J.E. (2014). Time-Resolved Serial Crystallography Captures High-Resolution Intermediates of Photoactive Yellow Protein. Science.

[6] Orville A.M. (2020). Recent Results in Time Resolved Serial Femtosecond Crystallography at XFELs. Curr. Opin. Struct. Biol..

[7] Barends T.R.M., Foucar L., Ardevol A., Nass K., Aquila A., Botha S., Doak R.B., Falahati K., Hartmann E., Hilpert M. (2015). Direct Observation of Ultrafast Collective Motions in CO Myoglobin upon Ligand Dissociation. Science.

[8] Monteiro D.C.F., Amoah E., Rogers C., Pearson A.R. (2021). Using Photocaging for Fast Time-Resolved Structural Biology Studies. Acta Crystallogr. D Struct. Biol..

[9] Smyth P., Jaho S., Williams L.J., Karras G., Fitzpatrick A., Thompson A.J., Battah S., Axford D., Horrell S., Lučić M. (2025). Time-Resolved Serial Synchrotron and Serial Femtosecond Crystallography of Heme Proteins Using Photocaged Nitric Oxide. IUCrJ.

[10] Park J., Nam K.H. (2024). Recent Chemical Mixing Devices for Time-Resolved Serial Femtosecond Crystallography. TrAC Trends Anal. Chem..

[11] Olmos J.L., Pandey S., Martin-Garcia J.M., Calvey G., Katz A., Knoska J., Kupitz C., Hunter M.S., Liang M., Oberthuer D. (2018). Enzyme Intermediates Captured “on the Fly” by Mix-and-Inject Serial Crystallography. BMC Biol..

[12] Wilson M.A. (2022). Mapping Enzyme Landscapes by Time-Resolved Crystallography with Synchrotron and X-Ray Free Electron Laser Light. Annu. Rev. Biophys..

[13] Kim J., Kim Y., Park J., Nam K.H., Cho Y. (2023). Structural Mechanism of *Escherichia Coli* Cyanase. Acta Crystallogr. D Struct. Biol..

[14] Chavas L.M.G., Gumprecht L., Chapman H.N. (2015). Possibilities for Serial Femtosecond Crystallography Sample Delivery at Future Light Sources. Struct. Dyn..

[15] Zhao F., Zhang B., Yan E., Sun B., Wang Z., He J., Yin D. (2019). A Guide to Sample Delivery Systems for Serial Crystallography. FEBS J..

[16] Grünbein M.L., Nass Kovacs G. (2019). Sample Delivery for Serial Crystallography at Free-Electron Lasers and Synchrotrons. Acta Crystallogr. D Struct. Biol..

[17] Cheng R. (2020). Towards an Optimal Sample Delivery Method for Serial Crystallography at XFEL. Crystals.

[18] Manna A., Doppler D., Sripati M.P., Sonker M., Ros A. (2025). Sample Delivery Methods for Protein X-Ray Crystallography with a Special Focus on Sample Consumption. Nat. Commun..

[19] Johansson L.C., Stauch B., Ishchenko A., Cherezov V. (2017). A Bright Future for Serial Femtosecond Crystallography with XFELs. Trends Biochem. Sci..

[20] White T.A., Kirian R.A., Martin A.V., Aquila A., Nass K., Barty A., Chapman H.N. (2012). *CrystFEL*: A Software Suite for Snapshot Serial Crystallography. J. Appl. Crystallogr..

[21] White T.A., Mariani V., Brehm W., Yefanov O., Barty A., Beyerlein K.R., Chervinskii F., Galli L., Gati C., Nakane T. (2016). Recent Developments in *CrystFEL*. J. Appl. Crystallogr..

[22] White T.A. (2019). Processing Serial Crystallography Data with *CrystFEL*: A Step-by-Step Guide. Acta Crystallogr. D Struct. Biol..

[23] Hattne J., Echols N., Tran R., Kern J., Gildea R.J., Brewster A.S., Alonso-Mori R., Glöckner C., Hellmich J., Laksmono H. (2014). Accurate Macromolecular Structures Using Minimal Measurements from X-Ray Free-Electron Lasers. Nat. Methods.

[24] DePonte D.P., Weierstall U., Schmidt K., Warner J., Starodub D., Spence J.C.H., Doak R.B. (2008). Gas Dynamic Virtual Nozzle for Generation of Microscopic Droplet Streams. J. Phys. D Appl. Phys..

[25] Davy B., Axford D., Beale J.H., Butryn A., Docker P., Ebrahim A., Leen G., Orville A.M., Owen R.L., Aller P. (2019). Reducing Sample Consumption for Serial Crystallography Using Acoustic Drop Ejection. J. Synchrotron Rad..

[26] Weierstall U., James D., Wang C., White T.A., Wang D., Liu W., Spence J.C.H., Bruce Doak R., Nelson G., Fromme P. (2014). Lipidic Cubic Phase Injector Facilitates Membrane Protein Serial Femtosecond Crystallography. Nat. Commun..

[27] Sugahara M., Mizohata E., Nango E., Suzuki M., Tanaka T., Masuda T., Tanaka R., Shimamura T., Tanaka Y., Suno C. (2015). Grease Matrix as a Versatile Carrier of Proteins for Serial Crystallography. Nat. Methods.

[28] Nam K.H. (2019). Sample Delivery Media for Serial Crystallography. Int. J. Mol. Sci..

[29] Calvey G.D., Katz A.M., Schaffer C.B., Pollack L. (2016). Mixing Injector Enables Time-Resolved Crystallography with High Hit Rate at X-Ray Free Electron Lasers. Struct. Dyn..

[30] Hunter M.S., Segelke B., Messerschmidt M., Williams G.J., Zatsepin N.A., Barty A., Benner W.H., Carlson D.B., Coleman M., Graf A. (2014). Fixed-Target Protein Serial Microcrystallography with an x-Ray Free Electron Laser. Sci. Rep..

[31] Mueller C., Marx A., Epp S.W., Zhong Y., Kuo A., Balo A.R., Soman J., Schotte F., Lemke H.T., Owen R.L. (2015). Fixed Target Matrix for Femtosecond Time-Resolved and in Situ Serial Micro-Crystallography. Struct. Dyn..

[32] Lee D., Baek S., Park J., Lee K., Kim J., Lee S.J., Chung W.K., Lee J.-L., Cho Y., Nam K.H. (2019). Nylon Mesh-Based Sample Holder for Fixed-Target Serial Femtosecond Crystallography. Sci. Rep..

[33] Butryn A., Simon P.S., Aller P., Hinchliffe P., Massad R.N., Leen G., Tooke C.L., Bogacz I., Kim I.-S., Bhowmick A. (2021). An On-Demand, Drop-on-Drop Method for Studying Enzyme Catalysis by Serial Crystallography. Nat. Commun..

[34] Mehrabi P., Schulz E.C., Agthe M., Horrell S., Bourenkov G., Von Stetten D., Leimkohl J.-P., Schikora H., Schneider T.R., Pearson A.R. (2019). Liquid Application Method for Time-Resolved Analyses by Serial Synchrotron Crystallography. Nat. Methods.

[35] Lee K., Kim J., Baek S., Park J., Park S., Lee J.-L., Chung W.K., Cho Y., Nam K.H. (2022). Combination of an Inject-and-Transfer System for Serial Femtosecond Crystallography. J. Appl. Crystallogr..

[36] Kang J., Shimazu Y., Luo F., Yamashita A., Tanaka T., Inubushi Y., Tono K., Nuemket N., Orville A.M., Iwata S. (2026). Compact Tape-Driven Sample Delivery System for Serial Femtosecond Crystallography. J. Appl. Crystallogr..

[37] Oghbaey S., Sarracini A., Ginn H.M., Pare-Labrosse O., Kuo A., Marx A., Epp S.W., Sherrell D.A., Eger B.T., Zhong Y. (2016). Fixed Target Combined with Spectral Mapping: Approaching 100% Hit Rates for Serial Crystallography. Acta Crystallogr. D Struct. Biol..

[38] Park J., Nam K.H. (2025). Application of Fixed-Target Microcrystal Delivery Systems for Serial Femtosecond Crystallography at PAL-XFEL. Analytica.

[39] Murray T.D., Lyubimov A.Y., Ogata C.M., Vo H., Uervirojnangkoorn M., Brunger A.T., Berger J.M. (2015). A High-Transparency, Micro-Patternable Chip for X-Ray Diffraction Analysis of Microcrystals under Native Growth Conditions. Acta Crystallogr. D Biol. Crystallogr..

[40] Doak R.B., Nass Kovacs G., Gorel A., Foucar L., Barends T.R.M., Grünbein M.L., Hilpert M., Kloos M., Roome C.M., Shoeman R.L. (2018). Crystallography on a Chip—Without the Chip: Sheet-on-Sheet Sandwich. Acta Crystallogr. D Struct. Biol..

[41] Doak R.B., Shoeman R.L., Gorel A., Niziński S., Barends T.R.M., Schlichting I. (2024). Sheet-on-Sheet Fixed Target Data Collection Devices for Serial Crystallography at Synchrotron and XFEL Sources. J. Appl. Crystallogr..

[42] Lee K., Lee D., Baek S., Park J., Lee S.J., Park S., Chung W.K., Lee J.-L., Cho H.-S., Cho Y. (2020). Viscous-Medium-Based Crystal Support in a Sample Holder for Fixed-Target Serial Femtosecond Crystallography. J. Appl. Crystallogr..

[43] Lee D., Park S., Lee K., Kim J., Park G., Nam K.H., Baek S., Chung W.K., Lee J.-L., Cho Y. (2020). Application of a High-Throughput Microcrystal Delivery System to Serial Femtosecond Crystallography. J. Appl. Crystallogr..

[44] Nam K.H., Kim J., Cho Y. (2021). Polyimide Mesh-Based Sample Holder with Irregular Crystal Mounting Holes for Fixed-Target Serial Crystallography. Sci. Rep..

[45] Manna A., Sonker M., Koh D., Steiger M., Ansari A., Hu H., Quereda-Moraleda I., Grieco A., Doppler D., De Sanctis D. (2024). Cyclic Olefin Copolymer-Based Fixed-Target Sample Delivery Device for Protein X-Ray Crystallography. Anal. Chem..

[46] Narayanasamy S.R., Shelby M.L., Chatterjee C., Zhou J., Rose S., Orlans J., Ghosh S., Cardenas A.M., Botha S., Gu K. (2025). Novel Polymer Fixed-Target Microfluidic Platforms with an Ultra-Thin Moisture Barrier for Serial Macromolecular Crystallography. bioRxiv.

[47] Park S.-Y., Choi H., Eo C., Cho Y., Nam K.H. (2020). Fixed-Target Serial Synchrotron Crystallography Using Nylon Mesh and Enclosed Film-Based Sample Holder. Crystals.

[48] Park J., Kim S., Nam K.-H., Kim B., Ko I.S. (2016). Current Status of the CXI Beamline at the PAL-XFEL. J. Korean Phys. Soc..

[49] Kang H.-S., Min C.-K., Heo H., Kim C., Yang H., Kim G., Nam I., Baek S.Y., Choi H.-J., Mun G. (2017). Hard X-Ray Free-Electron Laser with Femtosecond-Scale Timing Jitter. Nat. Photonics.

[50] Kim J., Kim H.-Y., Park J., Kim S., Kim S., Rah S., Lim J., Nam K.H. (2018). Focusing X-Ray Free-Electron Laser Pulses Using Kirkpatrick–Baez Mirrors at the NCI Hutch of the PAL-XFEL. J. Synchrotron Radiat..

[51] Mariani V., Morgan A., Yoon C.H., Lane T.J., White T.A., O’Grady C., Kuhn M., Aplin S., Koglin J., Barty A. (2016). *OnDA*: Online Data Analysis and Feedback for Serial X-Ray Imaging. J. Appl. Crystallogr..

[52] Nam K.H., Na S.-H. (2026). Serial Femtosecond Crystallography Data Processing at the Global Science Data Hub Center at KISTI. Sci. Rep..

[53] Barty A., Kirian R.A., Maia F.R.N.C., Hantke M., Yoon C.H., White T.A., Chapman H. (2014). *Cheetah*: Software for High-Throughput Reduction and Analysis of Serial Femtosecond X-Ray Diffraction Data. J. Appl. Crystallogr..

[54] Gevorkov Y., Yefanov O., Barty A., White T.A., Mariani V., Brehm W., Tolstikova A., Grigat R.-R., Chapman H.N. (2019). *XGANDALF*—Extended Gradient Descent Algorithm for Lattice Finding. Acta Crystallogr. A Found. Adv..

[55] Nam K.H., Park S., Park J. (2026). Effect of Crystal-to-Detector Distance Variations on Serial Femtosecond Crystallography Data Collected at PAL-XFEL. Crystals.

[56] Yefanov O., Mariani V., Gati C., White T.A., Chapman H.N., Barty A. (2015). Accurate Determination of Segmented X-Ray Detector Geometry. Opt. Express.

[57] Liebschner D., Afonine P.V., Baker M.L., Bunkóczi G., Chen V.B., Croll T.I., Hintze B., Hung L.-W., Jain S., McCoy A.J. (2019). Macromolecular Structure Determination Using X-Rays, Neutrons and Electrons: Recent Developments in *Phenix*. Acta Crystallogr. D Struct. Biol..

[58] Kim Y., Nam K.H. (2023). Fixed-Target Pink-Beam Serial Synchrotron Crystallography at Pohang Light Source II. Crystals.

[59] Emsley P., Cowtan K. (2004). *Coot*: Model-Building Tools for Molecular Graphics. Acta Crystallogr. D Biol. Crystallogr..

[60] Williams C.J., Headd J.J., Moriarty N.W., Prisant M.G., Videau L.L., Deis L.N., Verma V., Keedy D.A., Hintze B.J., Chen V.B. (2018). MolProbity: More and Better Reference Data for Improved All-atom Structure Validation. Protein Sci..

[61] Yang J., Jeon H.J., Park S., Park J., Jang S., Shin B., Bang K., Hawkes H.-J.K., Park S., Kim S. (2024). Structural Insights and Catalytic Mechanism of 3-Hydroxybutyryl-CoA Dehydrogenase from *Faecalibacterium Prausnitzii* A2-165. Int. J. Mol. Sci..

[62] Chung S., Kang M.-S., Alimbetov D.S., Mun G.-I., Yunn N.-O., Kim Y., Kim B.-G., Wie M., Lee E.A., Ra J.S. (2022). Regulation of BRCA1 Stability through the Tandem UBX Domains of Isoleucyl-tRNA Synthetase 1. Nat. Commun..

[63] Park J., Park S., Nam K.H. (2025). Preliminary Serial Femtosecond Crystallography Studies of Myoglobin from Equine Skeletal Muscle. Crystals.

[64] Gupta S., Dixit M., Sharma K., Saxena N.S. (2009). Mechanical Study of Metallized Polyethylene Terephthalate (PET) Films. Surf. Coat. Technol..

[65] Di Lorenzo M.L. (2024). Crystallization of Poly(Ethylene Terephthalate): A Review. Polymers.

[66] Lobley C.M.C., Sandy J., Sanchez-Weatherby J., Mazzorana M., Krojer T., Nowak R.P., Sorensen T.L. (2016). A Generic Protocol for Protein Crystal Dehydration Using the HC1b Humidity Controller. Acta Crystallogr. D Struct. Biol..

