# Supplementary material for: Polyethylene Terephthalate Film-Based Sample Holder for Fixed-Target Serial Femtosecond Crystallography
Source: Materials (Basel). 2026 Aug 14;19(16):3449. doi: 10.3390/ma19163449 (PMC13514868; doi:10.3390/ma19163449)
Supplement: Supplementary file 1 [file materials-19-03449-s001.zip › materials-4425990-supplementary.pdf]

Supplementary Data

Polyethylene terephthalate film-based sample holder for fixed-target serial femtosecond crystallography

Jaehyun Park [a,*], Sehan Park [a], Ki Hyun Nam [b,*]

[a] Pohang Accelerator Laboratory, Pohang University of Science and Technology, Pohang 37673, Republic of Korea.

[b] College of General Education, Kookmin University, Seoul 02707, Republic of Korea.

Electronic address: jaehyun.park@postech.ac.kr (J.P.), structure@kookmin.ac.kr (K.H.N.)

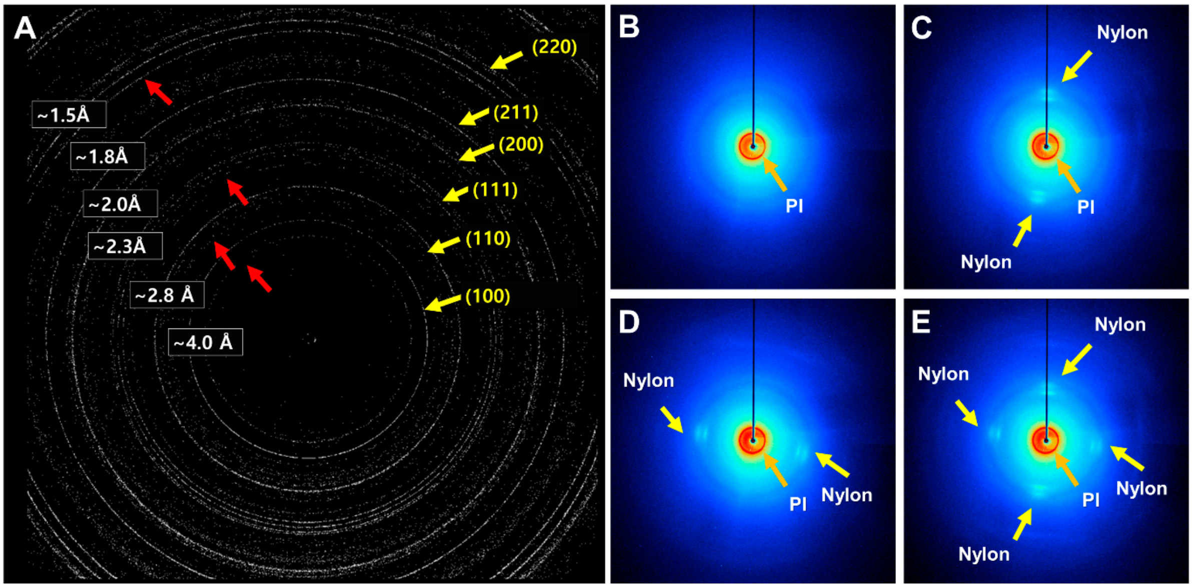

Supplementary Figure S1. Accumulated diffraction rings of BTO collected using a nylon mesh–based sample holder consisting of polyimide film and nylon mesh. (A) Accumulated BTO diffraction pattern. BTO and unwanted diffraction rings are indicated by yellow and red arrows, respectively. The diffraction rings at ~3.75/4.30 Å resolution were generated by the nylon mesh, whereas the diffraction ring from the polyimide at ~14 Å was not recorded due to the large beam stopper. XFEL scattering images of (B) nylon mesh pores, (C, D) nylon fibers, and (E) nylon intersections. The background scattering generated from polyimide and nylon mesh is indicated by orange and yellow arrows, respectively.

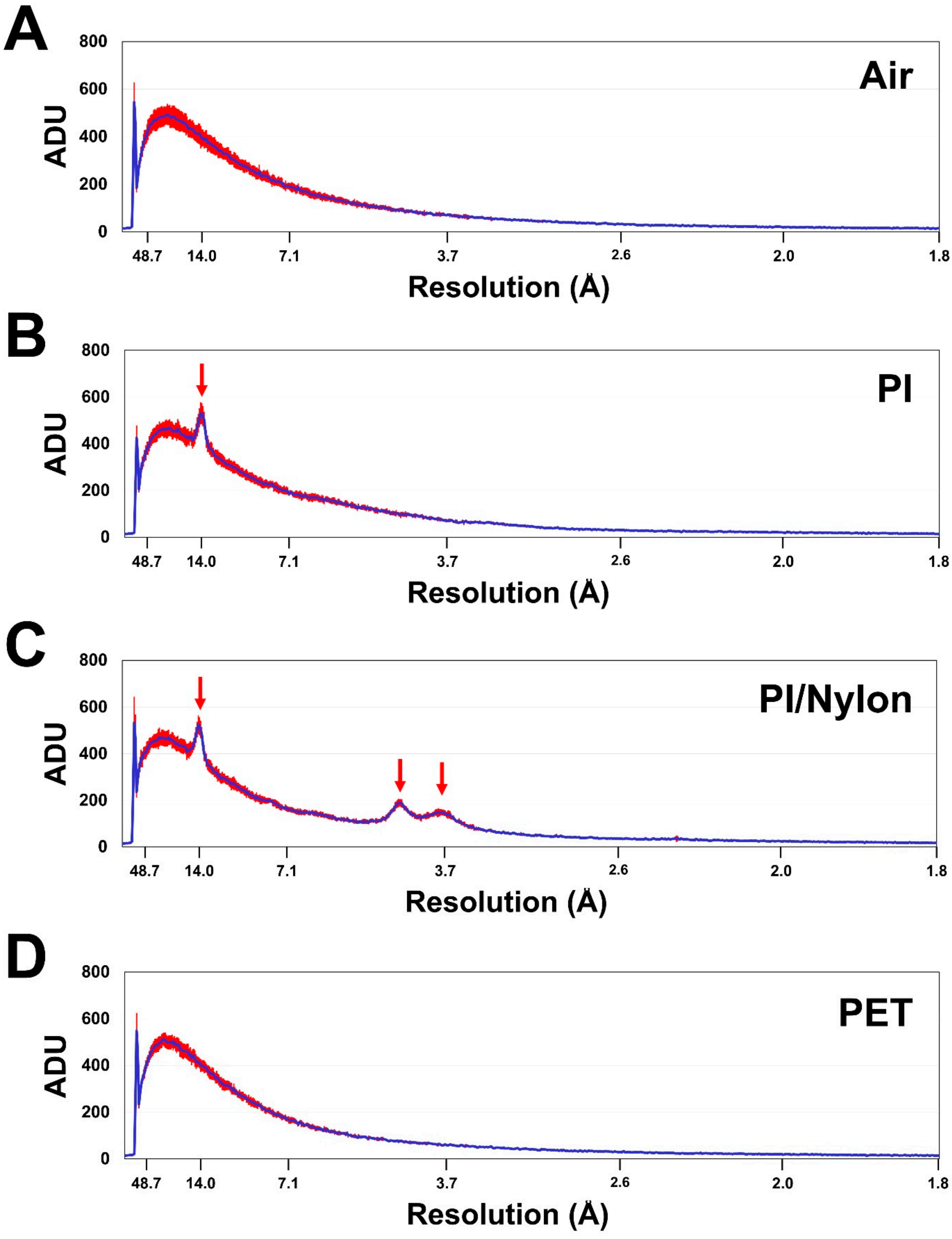

Supplementary Figure S2. XFEL background scattering intensities for (A) air, (B) polyimide film, (C) polyimide film with nylon mesh, and (D) PET film. Red traces indicate the standard deviation of the ADU values obtained from 10 independent diffraction images.

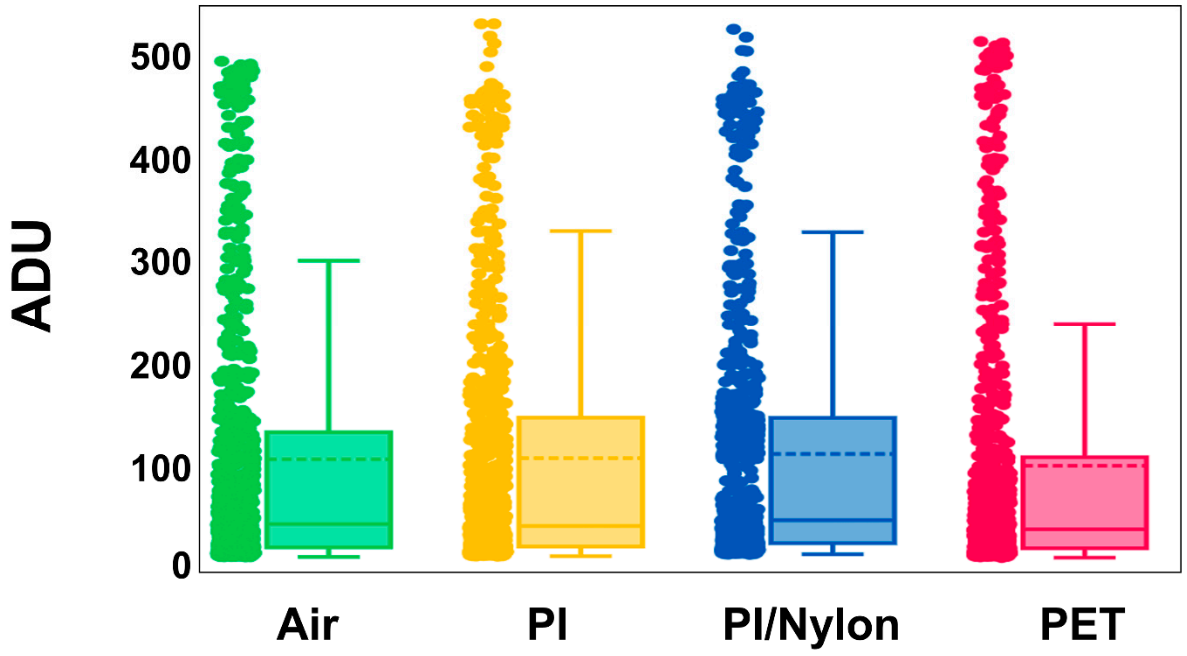

Supplementary Figure S3. Box plots of XFEL background scattering intensities (ADU) for air, polyimide (PI), polyimide with nylon mesh (PI/Nylon), and polyethylene terephthalate (PET). The ADU mean was indicated by dot line in the box plot. The mean ADU is indicated by a dotted line in the box plot

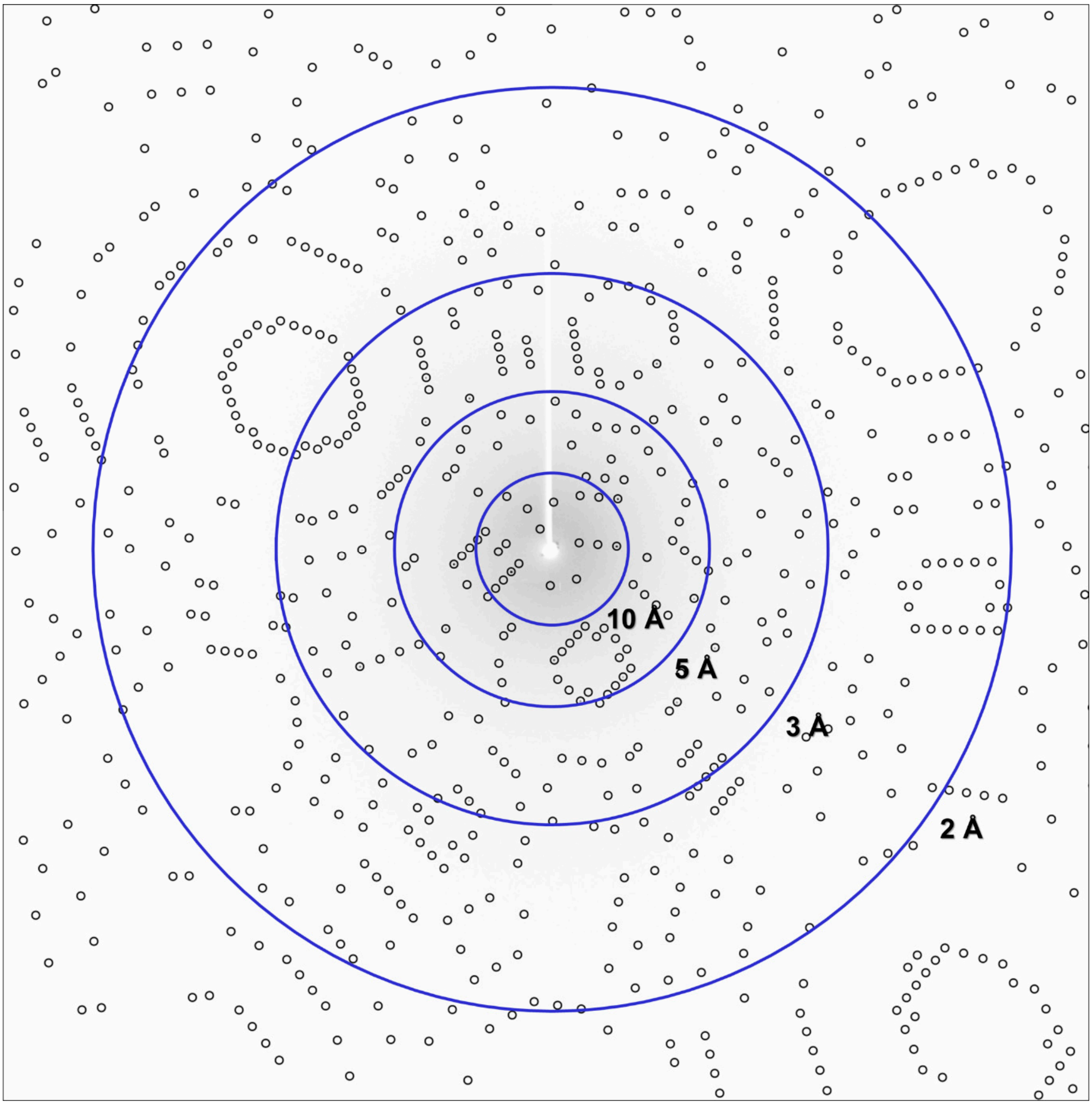

Supplementary Figure S4. Typical diffraction pattern of GI showing no significant background signal from the PET film-based sample holder.

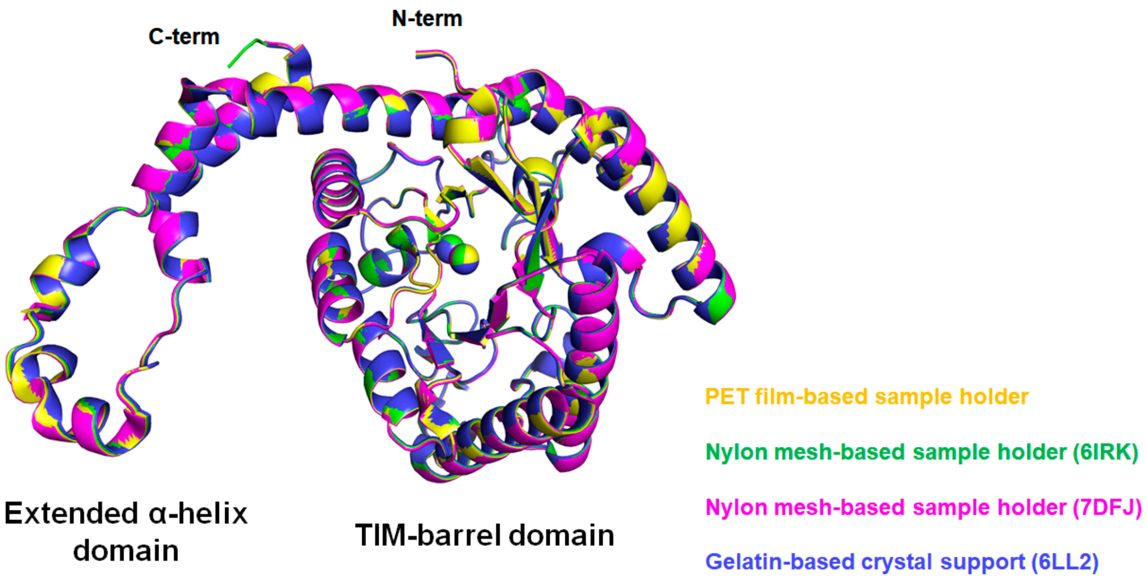

Supplementary Figure S5. Superimposition of GI structures obtained using the PET film-based sample holder (yellow) with previously reported structures obtained using the nylon mesh–based sample holder (PDB codes 6IRK and 7DFJ) and the gelatin-based crystal support (PDB code 6LL2).
